# The effect of sport-specific mindfulness on dispositional flow in collegiate athletes: the sequential mediating roles of cognitive reappraisal and sport confidence

**DOI:** 10.3389/fpsyg.2026.1859598

**Published:** 2026-09-24

**Authors:** Zhenyu Wang, Jingwen Zhang, Pengwei Song

**Affiliations:** 1 School of Physical Education and Health, Guangxi Science and Technology Normal University, Laibin, China; 2 Department of Physical Education, Graduate School, Keimyung University, Daegu, Republic of Korea; 3 Postdoctoral Research Station in Sports Science, Yangzhou University, Yangzhou, China

**Keywords:** cognitive reappraisal, collegiate athletes, dispositional flow, sport confidence, sport-specific mindfulness

## Abstract

**Objective:**

This study aimed to examine the association between sport-specific mindfulness and dispositional flow in collegiate athletes and to test whether cognitive reappraisal and sport confidence showed independent and sequential indirect effects in this association.

**Methods:**

A total of 583 collegiate athletes from universities in Guangxi, Hebei, Liaoning, and Tianjin, China, completed a cross-sectional questionnaire survey assessing sport-specific mindfulness, cognitive reappraisal, sport confidence, and dispositional flow. Confirmatory factor analysis, Pearson correlation analyses, regression analyses, and sequential mediation analysis using the PROCESS macro (version 5.0, Model 6) were conducted. Indirect effects were tested using 5,000 bias-corrected bootstrap samples, with sex, age group, and years of sport participation controlled.

**Results:**

Sport-specific mindfulness was significantly and positively correlated with cognitive reappraisal (*r* = 0.380, *p* < 0.001), sport confidence (*r* = 0.239, *p* < 0.001), and dispositional flow (*r* = 0.409, *p* < 0.001). After cognitive reappraisal and sport confidence were included in the model, the standardized direct effect of sport-specific mindfulness on dispositional flow remained significant (β = 0.267, 95% CI [0.190, 0.344]). The standardized total indirect effect was also significant (β = 0.144, 95% CI [0.100, 0.197]). Among the indirect pathways, cognitive reappraisal showed the largest standardized indirect effect (β = 0.113, 95% CI [0.077, 0.160]; 78.50% of the total indirect effect), followed by sport confidence (β = 0.019, 95% CI [0.005, 0.044]; 13.36%). The standardized sequential indirect effect through cognitive reappraisal and sport confidence was significant but small (β = 0.012, 95% CI [0.004, 0.026]; 8.14%).

**Conclusion:**

Sport-specific mindfulness was significantly and positively associated with dispositional flow among collegiate athletes. Cognitive reappraisal and sport confidence showed significant independent and sequential indirect effects in this association, with cognitive reappraisal accounting for the largest proportion of the total indirect effect. These findings indicate that cognitive reappraisal and sport confidence are important psychological factors for understanding the relationship between sport-specific mindfulness and dispositional flow among collegiate athletes.

## 1 Introduction

Sport-specific mindfulness refers to the application of mindfulness in competitive sport contexts. At its core, it involves athletes’ intentional regulation of attention toward present-moment experiences during training and competition, while approaching internal and external stimuli with openness, awareness, and acceptance (Cepeda Salas and Romero Carrasco, 2014; Gardner and Moore, 2012). In recent years, as mindfulness research has increasingly extended into sport settings, sport-specific mindfulness has been regarded as an important positive psychological resource associated with athletes’ psychological adaptation and sport performance (Contreras Pérez and Crobu, 2018; Birrer et al., 2023). Specifically, individuals with higher levels of sport-specific mindfulness tend to report better attentional control, more positive emotional experiences, and higher levels of dispositional flow (Jadhav, 2025; Guo, 2025). Although existing studies have provided preliminary evidence of a positive association between sport-specific mindfulness and dispositional flow, the psychological factors that may help explain this association remain insufficiently understood. Prior research has suggested that mindfulness is closely related to attentional regulation, cognitive control, and emotion regulation (Quaglia et al., 2019; Arora, 2025; Farb et al., 2012). Together, these findings point to the possible role of cognitive and emotional regulation in the association between sport-specific mindfulness and dispositional flow among collegiate athletes.

According to Monitor and Acceptance Theory, mindfulness involves present-moment monitoring and an accepting orientation, which may support adaptive cognitive and emotional regulation (Lindsay and Creswell, 2017; Simione and Saldarini, 2023). In sport contexts, this process may help athletes notice stressful events, negative emotions, and competitive pressure without reacting to them automatically (Mahalingam, 2022). By promoting decentering and non-judgmental awareness, sport-specific mindfulness may be associated with a greater tendency to reinterpret setbacks and challenges in more adaptive ways (Amemiya et al., 2019). Empirical evidence has also shown that mindfulness is positively associated with cognitive reappraisal, which may partly explain the link between mindfulness and positive psychological adaptation (Zhou et al., 2023). For collegiate athletes, cognitive reappraisal may support more constructive interpretations of training, competition, and academic demands and may be related to stronger task focus, perceived control, positive engagement, and dispositional flow (Stover et al., 2024; Regborn et al., 2025). Accordingly, cognitive reappraisal was considered a potential explanatory factor in the association between sport-specific mindfulness and dispositional flow.

At the same time, Conservation of Resources Theory proposes that people seek to acquire, maintain, and protect valued resources, which may accumulate over time (Radford, 2024). Sport-specific mindfulness can be viewed as a personal psychological resource because it supports attentional control, emotion regulation, and adaptation during training and competition (Sukmana et al., 2025). Sport confidence, defined as athletes’ positive beliefs about their athletic ability and task-coping capacity, is another positive psychological resource. It is related to engagement, perceived control, and persistence when facing challenges (Kuloor, 2024). From this perspective, sport-specific mindfulness may be associated with sport confidence through attention- and emotion-related resources. Consistent with this view, higher mindfulness has been linked to greater self-efficacy and confidence (Qin et al., 2022). Confidence may also help explain associations between mindfulness and positive psychological adaptation or sport performance (Chang et al., 2018). Among collegiate athletes, higher sport confidence may support engagement, task control, and expectations of success under academic and athletic pressure. These processes are closely related to dispositional flow. Therefore, sport confidence was considered another potential explanatory factor in this association.

Although previous studies have examined links among sport-specific mindfulness, cognitive reappraisal, sport confidence, and positive outcomes, much of this evidence comes from general student samples or broad athletic populations. Empirical work focusing on collegiate athletes remains relatively limited (Jadhav, 2025). Collegiate athletes occupy dual student and athlete roles and may face sustained tensions between academic and athletic demands (Lu et al., 2018). Existing findings may therefore not fully capture the psychological experiences of this group. In addition, prior research has often examined cognitive reappraisal and sport confidence separately. Less is known about whether they operate sequentially as indirect explanatory factors in the association between sport-specific mindfulness and dispositional flow. Social Cognitive Theory suggests that efficacy beliefs are shaped by cognitive processing styles and emotional states (Bandura, 2001). Adaptive cognitive appraisals may therefore relate to lower threat perception, stronger perceived control, and greater coping confidence. In sport contexts, cognitive reappraisal may be associated with more positive appraisals of stress and setbacks, stronger control beliefs, and higher sport confidence. Related studies have shown that cognitive reappraisal is positively associated with self-efficacy and confidence. Among athletes, adaptive emotion regulation is also often accompanied by greater competitive confidence (Robazza et al., 2023; Putri and Handayani, 2020). Therefore, the present study examined whether cognitive reappraisal and sport confidence independently and sequentially accounted for part of this association.

In the present study, these theoretical perspectives were integrated to explain the proposed sequential association among sport-specific mindfulness, cognitive reappraisal, sport confidence, and dispositional flow. Monitor and Acceptance Theory suggests that sport-specific mindfulness, through present-moment monitoring and acceptance, may be associated with more adaptive cognitive appraisal. Conservation of Resources Theory further frames mindfulness-related attentional and emotion-regulation capacities as personal psychological resources that may be linked to confidence-related resources in sport. Social Cognitive Theory helps clarify how appraisal processes are associated with efficacy-related beliefs, perceived control, and coping confidence. Taken together, these perspectives support a sequential model in which sport-specific mindfulness is associated with cognitive reappraisal, sport confidence, and dispositional flow. This theoretical ordering was used to guide model specification, while the cross-sectional design required cautious interpretation of the directionality among variables.

Based on this theoretical rationale, the present study examined the independent and sequential mediating roles of cognitive reappraisal and sport confidence in the association between sport-specific mindfulness and dispositional flow among collegiate athletes. This study adds empirical evidence from collegiate athletes and contributes to a deeper understanding of the psychological pathways through which mindful awareness, adaptive appraisal, and confidence-related self-beliefs are associated with dispositional flow. Accordingly, the following hypotheses were proposed, and a theoretical model was developed, as shown in Figure 1.

**FIGURE 1:**
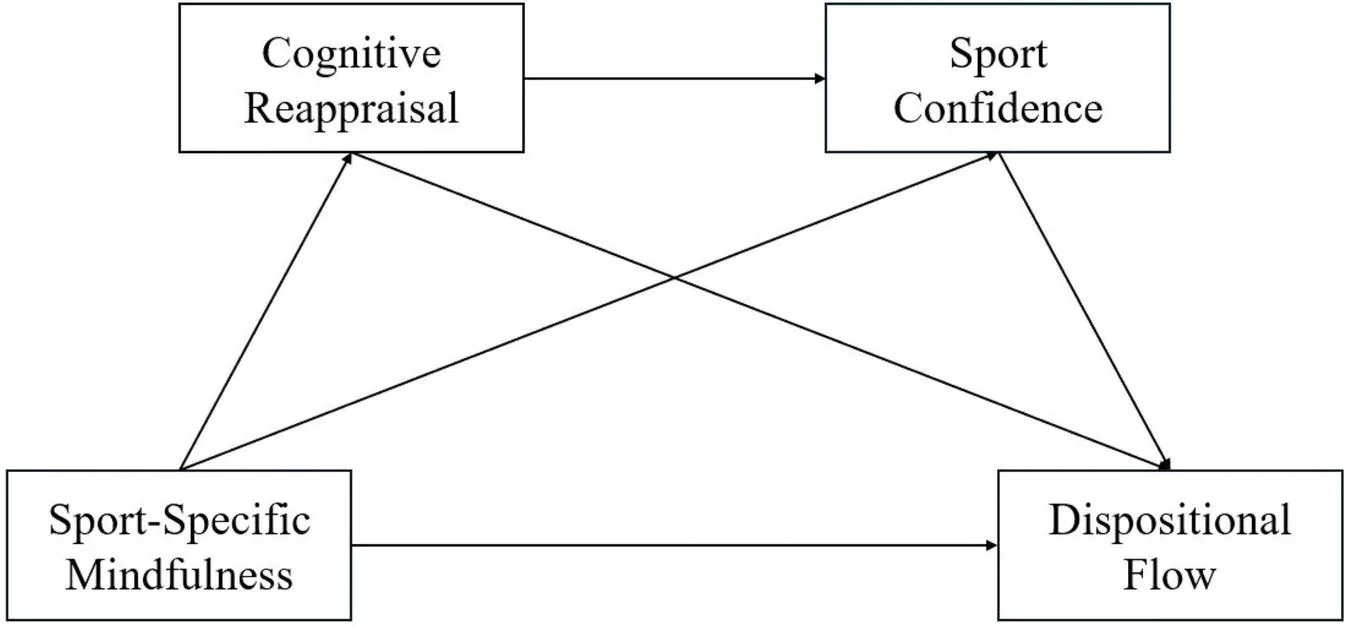
Hypothesized sequential mediation model.

*H1*: Sport-specific mindfulness is positively associated with dispositional flow in collegiate athletes.

*H2*: Cognitive reappraisal shows an indirect effect in the association between sport-specific mindfulness and dispositional flow in collegiate athletes.

*H3*: Sport confidence shows an indirect effect in the association between sport-specific mindfulness and dispositional flow in collegiate athletes.

*H4*: Cognitive reappraisal and sport confidence show a sequential indirect effect in the association between sport-specific mindfulness and dispositional flow in collegiate athletes.

## 2 Participants and methods

### 2.1 Participants

This study employed a cross-sectional questionnaire survey design. Data were collected between January 12, 2026, and March 5, 2026. A convenience sampling approach was used to recruit collegiate athletes with regular sport training experience from multiple universities in Guangxi Zhuang Autonomous Region, Hebei Province, Liaoning Province, and Tianjin, China. The inclusion criteria were as follows: (a) being enrolled as a college student, (b) having regular sport training experience, and (c) voluntarily agreeing to participate in the questionnaire survey. Questionnaires were excluded if they contained substantial missing responses, showed obvious response patterns, or were judged invalid during data screening. A total of 650 questionnaires were distributed. After excluding 67 invalid questionnaires, 583 valid questionnaires were retained, yielding a valid response rate of 89.69%.

Of the 583 participants, 352 were male (60.4%) and 231 were female (39.6%). In terms of age, 250 participants were between 18 and 20 years old (42.9%), 235 were between 21 and 23 years old (40.3%), and 98 were aged 24 years or older (16.8%). Regarding years of sport participation, 233 participants had participated in sport for 1–3 years (40.0%), 238 for 4–6 years (40.8%), 80 for 7–9 years (13.7%), and 32 for 10 years or more (5.5%).

### 2.2 Ethical considerations

This study was conducted in accordance with the principles of the Declaration of Helsinki and was approved by the Science and Technology Ethics Committee of Guangxi Science and Technology Normal University (Approval No. GSTNU-202511-001). All participants were collegiate athletes who voluntarily participated in the study and provided informed consent before completing the questionnaire. They retained the right to withdraw from the study at any stage.

To protect participant privacy, all questionnaire responses were collected and processed anonymously. Personal identifiers were not included in the dataset, and demographic information was recorded in grouped form, such as age group and years of sport participation. The anonymized data were used only for research purposes and were accessible only to the research team.

Through these procedures, the study ensured the protection of participants’ rights and privacy and complied with relevant ethical standards for human participant research.

### 2.3 Measures

Four psychometrically validated instruments were used to assess sport-specific mindfulness, cognitive reappraisal, sport confidence, and dispositional flow in collegiate athletes. All measures used Likert-type scales. Item responses were averaged to compute a mean score for each construct, with higher mean scores indicating higher levels of the corresponding construct.

#### 2.3.1 Sport-specific mindfulness

Sport-specific mindfulness was assessed using the revised Athlete Mindfulness Questionnaire (AMQ) developed by Zhang et al. (2017). The scale consisted of 16 items across three dimensions: present-moment attention (5 items), awareness (6 items), and acceptance (5 items). All items were rated on a 5-point Likert scale ranging from 1 (“never true”) to 5 (“always true”). Higher mean scores indicated higher levels of sport-specific mindfulness. In the present study, the scale demonstrated good reliability, with a Cronbach’s α of 0.911.

#### 2.3.2 Cognitive reappraisal

Cognitive reappraisal was measured using the Emotion Regulation Questionnaire (ERQ) developed by Gross and John (2003). The ERQ consisted of 10 items across two dimensions: cognitive reappraisal and expressive suppression. The cognitive reappraisal dimension included 6 items, whereas the expressive suppression dimension included 4 items. In the present study, only the 6 items from the cognitive reappraisal dimension were used and interpreted as an indicator of collegiate athletes’ general tendency to use cognitive reappraisal, rather than as a measure of sport-specific cognitive reappraisal during training or competition. Responses were rated on a 7-point Likert scale ranging from 1 (“strongly disagree”) to 7 (“strongly agree”). Higher mean scores indicated a greater tendency to use cognitive reappraisal as an emotion regulation strategy. This subscale showed good internal consistency, with a Cronbach’s α of 0.821.

#### 2.3.3 Sport confidence

Sport confidence was measured using the Sport-Confidence Inventory (SCI) developed by Vealey and Knight (2002). The scale consisted of 15 items covering three dimensions: confidence in physical skills and training, confidence in cognitive efficiency, and confidence in resilience. Responses were rated on a 7-point Likert scale ranging from 1 (“not at all capable”) to 7 (“completely certain”). Higher mean scores indicated higher levels of sport confidence. In the present study, the scale demonstrated good internal consistency, with a Cronbach’s α of 0.913.

#### 2.3.4 Dispositional flow

Dispositional flow was measured using the Chinese Dispositional Flow Scale-2 (CDFS-2), as revised by Liu et al. (2012). The scale consisted of 33 items across nine dimensions: challenge-skill balance, action-awareness merging, clear goals, unambiguous feedback, concentration on the task at hand, sense of control, loss of self-consciousness, transformation of time, and autotelic experience. Responses were rated on a 5-point Likert scale ranging from 1 (“never”) to 5 (“always”). Higher mean scores indicated higher levels of dispositional flow. In the present study, the scale showed good internal consistency, with a Cronbach’s α of 0.947.

### 2.4 Data analysis

Statistical analyses included confirmatory factor analysis, common method bias assessment, descriptive statistics, correlation analyses, regression analyses, and sequential mediation analysis. SPSS 26.0 was used for descriptive statistics, Cronbach’s α coefficients, demographic comparisons, Pearson correlation analyses, Harman’s single-factor test, and regression analyses. The PROCESS macro (Model 6, Version 5.0) was used for the sequential mediation analysis. Confirmatory factor analysis and the unmeasured latent method construct analysis were conducted in R 4.5.1 using the lavaan package, and CR and AVE were calculated from standardized factor loadings. Before the main analyses, a four-factor confirmatory factor analysis was conducted to examine the measurement structure of sport-specific mindfulness, cognitive reappraisal, sport confidence, and dispositional flow. The CFA was conducted using the 18 dimension-level indicators for the four latent constructs. Composite reliability (CR) and average variance extracted (AVE) were calculated to evaluate construct reliability and convergent validity. Common method bias was then assessed using Harman’s single-factor test and an unmeasured latent method construct (ULMC) approach. In the ULMC analysis, a common method factor was added to the baseline four-factor CFA model, and model fit indices and standardized factor loadings were compared before and after including the common method factor.

Descriptive statistics and Cronbach’s α coefficients were computed for all study variables. Differences in the study variables across sex, age group, and years of sport participation were examined using independent-samples *t*-tests for sex and one-way analyses of variance for age group and years of sport participation. Pearson correlation analyses were then conducted to examine the associations among sport-specific mindfulness, cognitive reappraisal, sport confidence, and dispositional flow. The proposed sequential mediation model was tested with sport-specific mindfulness as the independent variable, dispositional flow as the dependent variable, and cognitive reappraisal and sport confidence as the mediators. Sex, age group, and years of sport participation were included as control variables. Mean item scores were used in the descriptive and correlation analyses. Before the regression and PROCESS analyses, the four continuous study variables were standardized. Accordingly, all reported path coefficients and total, direct, and indirect effects are standardized. To further evaluate the proposed model specification, sensitivity analyses were conducted by testing competing models, including a reversed mediator-order model and reverse models with dispositional flow specified as the predictor. A bias-corrected bootstrap analysis with 5,000 resamples was used to test the significance of the indirect effects. Indirect effects were considered significant when the 95% confidence interval did not include zero. All statistical tests were two-tailed, and the significance level was set at *p* < 0.05.

## 3 Results

### 3.1 Measurement model and construct validity

A four-factor CFA was conducted using 18 indicators, including dimension-level indicators for sport-specific mindfulness, sport confidence, and dispositional flow, and three two-item parcels for cognitive reappraisal. The results of the measurement model are presented in Table 1. The model showed good fit to the data, χ^2^(129) = 189.418, χ^2^/df = 1.468, CFI = 0.992, TLI = 0.990, RMSEA = 0.028, and SRMR = 0.023. Standardized factor loadings ranged from 0.856 to 0.891 for sport-specific mindfulness, 0.736–0.820 for cognitive reappraisal, 0.788–0.832 for sport confidence, and 0.791–0.846 for dispositional flow. CR values ranged from 0.818 to 0.950, and AVE values ranged from 0.601 to 0.763. These results provided evidence of acceptable composite reliability and convergent validity for the four constructs in this sample.

**TABLE 1:** Reliability and convergent validity of the measurement model.

Construct	Standardized factor loading range	CR	AVE
Sport-specific mindfulness	0.856–0.891	0.906	0.763
Cognitive reappraisal	0.736–0.820	0.818	0.601
Sport confidence	0.788–0.832	0.851	0.655
Dispositional flow	0.791–0.846	0.950	0.680

CR, composite reliability; AVE, average variance extracted.

### 3.2 Assessment of common method bias

Common method bias was evaluated using Harman’s single-factor test and an unmeasured latent method construct (ULMC) sensitivity analysis. The results of these analyses are presented in Table 2. In Harman’s single-factor test, the 70 item-level indicators yielded 10 factors with eigenvalues greater than 1, and the first unrotated factor explained 23.181% of the total variance, which was below the commonly used 40% threshold. The baseline four-factor CFA showed good fit to the data, χ^2^(129) = 189.418, χ^2^/df = 1.468, CFI = 0.992, TLI = 0.990, RMSEA = 0.028, and SRMR = 0.023. After adding a common method factor to the 18 indicators, the model also showed good fit, χ^2^(111) = 156.897, χ^2^/df = 1.413, CFI = 0.994, TLI = 0.991, RMSEA = 0.027, and SRMR = 0.020. The changes in fit indices were small, suggesting that the inclusion of a common method factor did not meaningfully improve the model. Taken together, the Harman test and the ULMC sensitivity analysis did not indicate substantial common method bias in the present data.

**TABLE 2:** Comparison of model fit before and after adding the common method factor.

Model	χ^2^	Df	χ^2^/df	CFI	TLI	RMSEA	SRMR
Baseline CFA	189.418	129	1.468	0.992	0.990	0.028	0.023
ULMC model	156.897	111	1.413	0.994	0.991	0.027	0.020
Difference	—	—	—	0.002	0.001	−0.001	−0.003

ULMC, unmeasured latent method construct. Difference values were calculated as the ULMC model minus the baseline CFA model.

### 3.3 Descriptive statistics and demographic comparisons

Descriptive statistics were calculated for sport-specific mindfulness, cognitive reappraisal, sport confidence, and dispositional flow. Group differences in the study variables by sex, age group, and years of sport participation were examined using independent-samples *t*-tests and one-way analyses of variance. The results are presented in Table 3. Significant sex differences were found in cognitive reappraisal and sport confidence, whereas no significant sex differences were observed in sport-specific mindfulness or dispositional flow. No significant differences across age groups were found for any of the four variables (*p* > 0.05). Significant differences across years of sport participation were found in sport-specific mindfulness and dispositional flow, but not in cognitive reappraisal or sport confidence. Tukey *post-hoc* comparisons showed that participants with 4–6 years of sport participation reported higher sport-specific mindfulness than those with 1–3 years of participation. For dispositional flow, participants with 7–9 years of sport participation scored lower than those with 4–6 years of participation. The effect sizes for the significant differences in sport-specific mindfulness and dispositional flow across years of sport participation were small (η^2^ = 0.024–0.037).

**TABLE 3:** Descriptive statistics and demographic differences in study variables.

Variable	Group	n	Sport-specific mindfulness	Cognitive reappraisal	Sport confidence	Dispositional flow
Sex	Male	352	4.11 ± 0.62	5.26 ± 0.87	4.96 ± 0.88	3.84 ± 0.65
	Female	231	4.05 ± 0.56	5.04 ± 0.86	4.76 ± 0.94	3.79 ± 0.62
	*t*		1.159	3.008**	2.674**	0.909
	*p*		0.247	0.003	0.008	0.364
Age	18–20 years	250	4.03 ± 0.70	5.18 ± 0.92	4.92 ± 0.92	3.78 ± 0.68
	21–23 years	235	4.13 ± 0.52	5.19 ± 0.82	4.86 ± 0.93	3.85 ± 0.63
	24 years and above	98	4.12 ± 0.46	5.09 ± 0.86	4.83 ± 0.86	3.83 ± 0.55
	*F*		1.780	0.565	0.354	0.592
	*p*		0.170	0.569	0.702	0.553
Years of sport participation	1–3 years	233	3.96 ± 0.73	5.14 ± 0.94	4.90 ± 0.92	3.77 ± 0.67
	4–6 years	238	4.21 ± 0.40	5.26 ± 0.78	4.85 ± 0.87	3.91 ± 0.55
	7–9 years	80	4.06 ± 0.61	5.05 ± 0.92	4.86 ± 1.05	3.63 ± 0.80
	10 years and above	32	4.18 ± 0.56	5.05 ± 0.82	5.03 ± 0.75	3.94 ± 0.43
	*F*		7.372***	1.633	0.423	4.828**
	*p*		< 0.001	0.181	0.736	0.003

Data are presented as M ± SD. *Post-hoc* comparisons were conducted using Tukey’s HSD test. Effect sizes for ANOVA were indexed by η^2^. **p < 0.01, ***p < 0.001.

### 3.4 Correlation analyses of the study variables

Pearson correlation analyses were conducted to examine the associations among the study variables, and the results are presented in Table 4. Sport-specific mindfulness was positively correlated with cognitive reappraisal (*r* = 0.380), sport confidence (*r* = 0.239), and dispositional flow (*r* = 0.409), all *p* < 0.001. Cognitive reappraisal was positively correlated with sport confidence (*r* = 0.301) and dispositional flow (*r* = 0.431), both *p* < 0.001. In addition, sport confidence was positively correlated with dispositional flow (*r* = 0.278, *p* < 0.001).

**TABLE 4:** Descriptive statistics and correlations among the study variables.

Variable	*M*	SD	1	2	3	4
1. Sport-specific mindfulness	4.09	0.60	1	1	1	1
2. Cognitive reappraisal	5.17	0.87	0.380***			
3. Sport confidence	4.88	0.91	0.239***	0.301***		
4. Dispositional flow	3.82	0.64	0.409***	0.431***	0.278***	

***p < 0.001.

### 3.5 Regression analyses for the sequential mediation model

After controlling for sex, age group, and years of sport participation, regression analyses were conducted to estimate the standardized path coefficients in the proposed sequential mediation model. The results are presented in Table 5. Sport-specific mindfulness was significantly and positively associated with dispositional flow (β = 0.411, *p* < 0.001), which was consistent with H1. Sport-specific mindfulness was significantly and positively associated with cognitive reappraisal (β = 0.384, *p* < 0.001), and cognitive reappraisal was significantly and positively associated with dispositional flow (β = 0.293, *p* < 0.001). In addition, sport-specific mindfulness was significantly and positively associated with sport confidence (β = 0.148, *p* < 0.001), and sport confidence was significantly and positively associated with dispositional flow (β = 0.130, *p* < 0.001). Cognitive reappraisal was also significantly and positively associated with sport confidence (β = 0.236, *p* < 0.001). After cognitive reappraisal and sport confidence were included in the model, the direct association between sport-specific mindfulness and dispositional flow remained significant (β = 0.267, *p* < 0.001), indicating a pattern consistent with partial statistical mediation in the tested model.

**TABLE 5:** Results of the regression analyses.

Predictor variable	Dispositional flow (total effect model)			Cognitive reappraisal			Sport confidence			Dispositional flow (direct effect model)		
	β	SE	*t*	β	SE	*t*	β	SE	*t*	β	SE	*t*
Constant	0.097	0.156	0.622	0.500	0.156	3.196**	0.268	0.162	1.656	−0.100	0.148	−0.677
Sex	−0.040	0.078	−0.517	−0.205	0.079	−2.607**	−0.144	0.081	−1.767	0.045	0.074	0.602
Age group	0.023	0.053	0.432	−0.047	0.053	−0.872	−0.038	0.055	−0.691	0.043	0.050	0.861
Years of sport participation	−0.043	0.045	−0.967	−0.072	0.045	−1.589	−0.001	0.047	−0.015	−0.020	0.042	−0.462
Sport-specific mindfulness	0.411	0.038	10.730***	0.384	0.038	9.995***	0.148	0.043	3.459***	0.267	0.039	6.799***
Cognitive reappraisal							0.236	0.043	5.511***	0.293	0.040	7.367***
Sport confidence										0.130	0.038	3.427***
R	0.411			0.401			0.339			0.521		
R^2^	0.169			0.161			0.115			0.272		
F	29.332***			27.720***			15.017***			35.804***		

Sex, age group, and years of sport participation were included as control variables. The total effect model refers to the regression model testing the effect of sport-specific mindfulness on dispositional flow before the mediators were entered. The direct effect model refers to the regression model testing the effect of sport-specific mindfulness on dispositional flow after cognitive reappraisal and sport confidence were entered simultaneously. β = standardized regression coefficient; SE = standard error. **p < 0.01, ***p < 0.001.

### 3.6 Test of the sequential mediation effects

A bootstrap procedure was used to test the indirect effects in the proposed sequential mediation model. The results are presented in Figure 2 and Table 6. The standardized total indirect effect was significant (β = 0.144, 95% CI [0.100, 0.197]). Specifically, the standardized indirect effect through cognitive reappraisal was significant (β = 0.113, 95% CI [0.077, 0.160]), accounting for 78.50% of the total indirect effect. The standardized indirect effect through sport confidence was also significant (β = 0.019, 95% CI [0.005, 0.044]), accounting for 13.36% of the total indirect effect. In addition, the standardized sequential indirect effect through cognitive reappraisal and sport confidence was statistically significant, although it was small in magnitude (β = 0.012, 95% CI [0.004, 0.026]), accounting for 8.14% of the total indirect effect. The 95% confidence intervals for all indirect effects did not include zero, indicating significant specific and sequential indirect effects within the tested model. These results were consistent with H2, H3, and H4.

**FIGURE 2:**
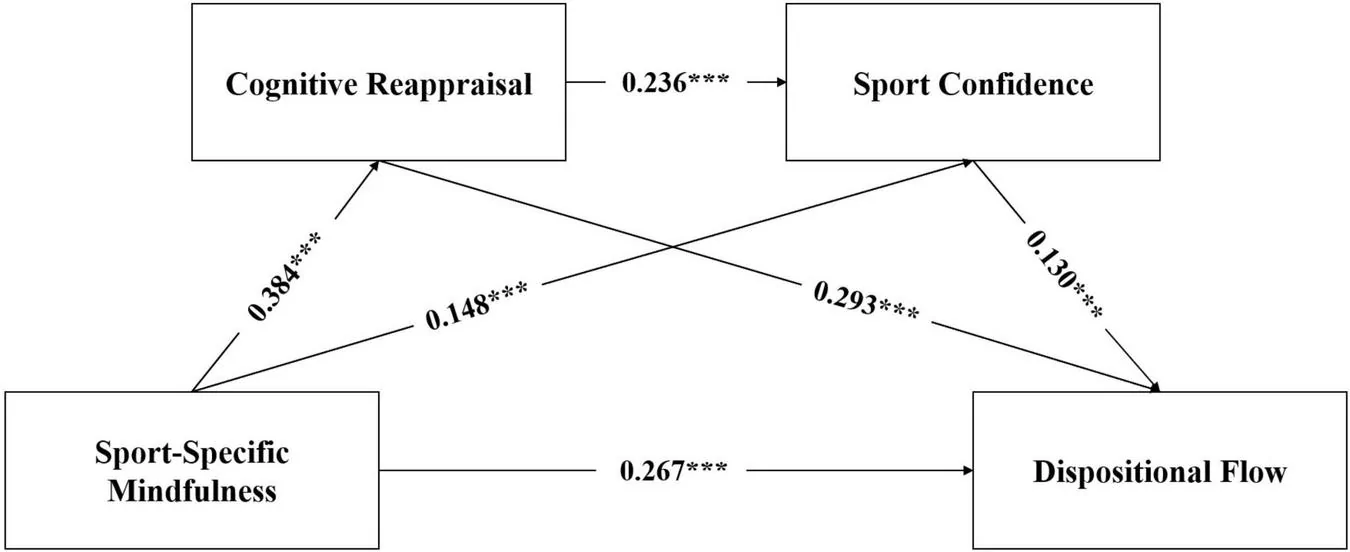
Standardized path coefficients of the tested sequential mediation model. Values are standardized regression coefficients. Sex, age group, and years of sport participation were included as control variables. ***p < 0.001.

**TABLE 6:** Bootstrap results for the sequential mediation effects.

Effect type	Standardized effect (β)	Boot SE	Bootstrap 95% CI	Proportion of the total indirect effect
Total effect	0.411	0.038	[0.336, 0.486]	–
Direct effect	0.267	0.039	[0.190, 0.344]	–
Total indirect effect	0.144	0.025	[0.100, 0.197]	100%
Sport-specific mindfulness → cognitive reappraisal → dispositional flow	0.113	0.021	[0.077, 0.160]	78.50%
Sport-specific mindfulness → sport confidence → dispositional flow	0.019	0.010	[0.005, 0.044]	13.36%
Sport-specific mindfulness → cognitive reappraisal → sport confidence → dispositional flow	0.012	0.006	[0.004, 0.026]	8.14%

Bootstrap resampling was conducted with 5,000 samples. Effect estimates and bootstrap standard errors are standardized. Bias-corrected 95% bootstrap confidence intervals are reported for the indirect effects. The proportion was calculated as the ratio of each specific indirect effect to the total indirect effect. β = standardized effect; Boot SE = bootstrap standard error.

We further conducted sensitivity analyses to examine whether the indirect-effect pattern changed across three alternative PROCESS Model 6 specifications. These analyses were intended to assess the robustness of the indirect-effect estimates under different model specifications, rather than to establish the temporal or causal ordering of the variables. As shown in Supplementary Table S1, when the order of cognitive reappraisal and sport confidence was reversed while sport-specific mindfulness remained the predictor and dispositional flow remained the outcome, the total indirect effect remained 0.144. However, the decomposition of the specific indirect effects changed. In the proposed model, the indirect effects through cognitive reappraisal alone, sport confidence alone, and the sequential pathway were 0.113, 0.019, and 0.012, respectively. In the reversed mediator-order model, the indirect effects through sport confidence alone, cognitive reappraisal alone, and the reversed sequential pathway were 0.031, 0.098, and 0.015, respectively. When dispositional flow was specified as the predictor and sport-specific mindfulness as the outcome, the total indirect effect was 0.126, with specific indirect effects of 0.101, 0.017, and 0.008. When both the predictor-outcome direction and the mediator order were reversed, the total indirect effect was also 0.126, with specific indirect effects of 0.025, 0.089, and 0.012. These results indicate that the overall indirect-effect estimates were broadly comparable across competing specifications, whereas the distribution of specific indirect effects varied according to mediator order and predictor-outcome specification. The broadly comparable indirect-effect patterns across these cross-sectional specifications are consistent with the known limitation that cross-sectional mediation models cannot establish temporal ordering. Therefore, we interpreted the proposed model as a theoretically guided representation of the observed associations, while the temporal sequence among sport-specific mindfulness, cognitive reappraisal, sport confidence, and dispositional flow requires further examination in longitudinal or experimental studies.

## 4 Discussion

The present study examined the relationship between sport-specific mindfulness and dispositional flow in collegiate athletes, with a focus on the mediating roles of cognitive reappraisal and sport confidence. Overall, the findings suggest that sport-specific mindfulness is positively associated with dispositional flow and that this association may be partly explained by cognitive reappraisal and sport confidence. The independent indirect effects were more pronounced than the sequential indirect effect, which was statistically significant but modest. These findings provide preliminary evidence for psychological processes that may link sport-specific mindfulness with dispositional flow in collegiate athletes.

### 4.1 The relationship between sport-specific mindfulness and dispositional flow

The results of the present study showed that sport-specific mindfulness was significantly and positively associated with dispositional flow in collegiate athletes, which was consistent with H1. This finding aligns with Guo (2025), who reported a positive association between mindfulness, sport-related experiences, and flow. It also supports the relevance of Monitor and Acceptance Theory for understanding psychological experiences in competitive sport settings. According to this theory, mindfulness may support attentional regulation and adaptive emotional responses through sustained awareness of present-moment experience and non-judgmental acceptance, thereby helping to reduce the disruptive influence of automatic negative cognitions and avoidance tendencies on behavioral performance (Wenzel et al., 2021). From this perspective, sport-specific mindfulness may function as a sport-related psychological resource that helps athletes maintain attention on movement execution, task goals, and immediate feedback during training and competition (Hadash et al., 2026). It may also help athletes respond to environmental fluctuations, technical errors, and internal emotional changes with a more open and accepting attitude, thereby helping to reduce the interference of state anxiety, attentional distraction, and negative self-evaluation during sport participation (Noetel et al., 2017). Stable attentional engagement and a positive psychological state may be associated with a stronger sense of control and immersion, both of which are central features of dispositional flow. The participants in the present study were collegiate athletes who faced both academic demands and regular sport training and were therefore exposed to multiple role-related stressors. In such circumstances, their attentional resources may be more susceptible to task-irrelevant stimuli and negative emotions (Xu et al., 2025; Young et al., 2023). Sport-specific mindfulness may therefore be particularly relevant for this population, as it may help athletes enter a focused state more readily and maintain perceived control during training and competition through the regulation of attention and emotion.

Taken together, sport-specific mindfulness appears to be an important psychological factor associated with dispositional flow in collegiate athletes. Within the tested model, cognitive reappraisal and sport confidence further contributed to explaining this association. These findings suggest that sport-specific mindfulness may be a relevant focus for future sport psychology research and psychological support for collegiate athletes.

### 4.2 The mediating role of cognitive reappraisal

The mediation analysis showed a significant indirect effect through cognitive reappraisal in the relationship between sport-specific mindfulness and dispositional flow, which was consistent with H2. This finding supports the relevance of Monitor and Acceptance Theory in competitive sport contexts, particularly the view that mindfulness may be associated with more adaptive cognitive and emotional regulation processes. Cognitive reappraisal, as an adaptive emotion regulation strategy, may therefore be an important psychological process underlying positive sport-related experiences among collegiate athletes (Baumgartner et al., 2022). This interpretation is consistent with Pidgeon et al. (2014), who identified cognitive reappraisal as an important cognitive regulatory process linking mindfulness with positive psychological adaptation. It is also consistent with Robazza et al. (2023), who reported that cognitive reappraisal is closely associated with athletes’ positive emotional experiences, perceived control, task focus, and adaptive coping under training and competitive pressure.

More specifically, collegiate athletes with higher levels of sport-specific mindfulness may approach stressful events, technical errors, and emotional fluctuations during training and competition with greater awareness and non-judgmental acceptance. This may reduce automatic involvement with negative stimuli, ruminative thinking, and negative cognitive bias, thereby helping athletes reframe competitive situations in a more positive and flexible manner. In addition, a greater general tendency to use cognitive reappraisal may be relevant to how collegiate athletes interpret training load, competitive pressure, and temporary setbacks as manageable and controllable developmental challenges. Such adaptive appraisal tendencies may be associated with less emotional disruption during sport tasks and with greater task focus, perceived control, and positive emotional experience, all of which are closely related to dispositional flow.

Taken together, cognitive reappraisal may help explain how sport-specific mindfulness is associated with dispositional flow among collegiate athletes. Previous studies have largely explained flow in sport from the perspective of mindfulness itself, particularly its attentional control function and its association with positive emotions. By incorporating cognitive reappraisal as a mediator, the present study adds evidence that cognitive-emotional regulation may be relevant to the association between sport-specific mindfulness and dispositional flow among collegiate athletes (Cox et al., 2020; Scott-Hamilton et al., 2016).

The interpretation of this pathway should also take into account the level at which cognitive reappraisal was measured. In the present study, cognitive reappraisal was assessed as a general emotion-regulation tendency, whereas sport-specific mindfulness, sport confidence, and dispositional flow were assessed in sport-related contexts. This difference in measurement context may have attenuated the associations involving cognitive reappraisal, particularly the associations of sport-specific mindfulness with cognitive reappraisal and of cognitive reappraisal with sport confidence. A general tendency to use cognitive reappraisal may not fully capture how athletes reinterpret sport-specific experiences such as training stress, technical errors, competitive pressure, or performance setbacks. Accordingly, the indirect effects involving cognitive reappraisal may represent conservative estimates of appraisal-related regulation in sport contexts. Future studies using sport-specific measures of cognitive reappraisal could provide a more precise test of this pathway.

### 4.3 The mediating role of sport confidence

The mediation analysis showed a significant indirect effect through sport confidence in the relationship between sport-specific mindfulness and dispositional flow, which was consistent with H3. Collegiate athletes with higher levels of sport-specific mindfulness tended to report higher sport confidence, and higher sport confidence was, in turn, associated with higher dispositional flow. Rather than indicating a causal developmental sequence, this pattern suggests that sport confidence may serve as an important psychological link within the tested mediation model. This interpretation is consistent with previous research indicating that mindfulness and acceptance-based approaches are related to self-efficacy, confidence, and adaptive psychological functioning in sport contexts (Noetel et al., 2017). In addition, Gilson (2010) and Jekauc et al. (2025) reported that sport confidence is closely related to athletes’ perceived control, engagement, expectations of success, athletic performance, and positive psychological experiences in training and competition. These confidence-related experiences overlap with several core features of dispositional flow, particularly perceived control, task engagement, and positive involvement in sport.

Furthermore, the present findings are broadly consistent with Conservation of Resources Theory. According to this perspective, positive psychological resources may coexist and accumulate, and initial psychological resources may be associated with the development of further adaptive resources. Within the present study, sport-specific mindfulness may be understood as a psychological resource related to attentional control, emotional awareness, and acceptance in sport contexts, whereas sport confidence may represent a more specific self-belief resource concerning athletes’ perceived ability to cope with task demands (Chang et al., 2018). This resource-based interpretation helps explain the observed indirect association between sport-specific mindfulness and dispositional flow through sport confidence, while remaining consistent with the cross-sectional nature of the data.

### 4.4 The sequential mediating roles of cognitive reappraisal and sport confidence

The present study also found a statistically significant sequential indirect effect of cognitive reappraisal and sport confidence in the association between sport-specific mindfulness and dispositional flow, which was consistent with H4. However, this sequential indirect effect was small in magnitude, accounting for only 8.14% of the total indirect effect. This pathway may therefore be interpreted as a modest but theoretically relevant component of the tested model. This pattern is broadly consistent with the findings reported by Rogowska and Tataruch (2024).

Within the tested model, higher sport-specific mindfulness was associated with higher cognitive reappraisal, higher cognitive reappraisal was associated with higher sport confidence, and higher sport confidence was associated with higher dispositional flow. This pattern is compatible with Social Cognitive Theory, which emphasizes that efficacy beliefs are related to how individuals interpret internal states and situational demands. Adaptive cognitive appraisal may be associated with lower perceived threat, stronger perceived control, and greater confidence in coping with challenges (Usher and Morris, 2023). From this perspective, cognitive reappraisal may help explain how collegiate athletes interpret stressors, setbacks, and competitive demands in a more manageable way, whereas sport confidence may represent a self-belief resource linked to perceived control and engagement during sport participation. The observed sequential indirect effect therefore suggests that cognitive-emotional regulation and sport-related self-beliefs may jointly help account for the association between sport-specific mindfulness and dispositional flow.

In terms of relative effect size, the independent indirect effect through cognitive reappraisal accounted for the largest proportion of the total indirect effect (78.50%), suggesting that cognitive reappraisal may be a particularly important explanatory pathway in the tested model. The comparatively smaller sequential indirect effect indicates that the pathway from cognitive reappraisal to sport confidence warrants further examination in longitudinal or intervention studies. From an applied perspective, the present findings suggest that mindfulness, cognitive reappraisal, and sport confidence could be considered together in future sport psychology programs for collegiate athletes. However, given the cross-sectional design, these applied implications should be interpreted cautiously. Future experimental research is needed to test whether mindfulness-oriented training, cognitive reappraisal strategies, and confidence-building components may jointly contribute to dispositional flow in collegiate athletes. Taken together, the theoretical perspectives discussed above converge on a self-regulatory interpretation in which present-moment awareness, adaptive appraisal, and confidence-related self-beliefs are interrelated psychological factors associated with dispositional flow.

## 5 Conclusions and limitations

### 5.1 Conclusion

(1) Sport-specific mindfulness, cognitive reappraisal, sport confidence, and dispositional flow were significantly and positively correlated among collegiate athletes.

(2) Sport-specific mindfulness was directly and indirectly associated with dispositional flow. Specifically, sport-specific mindfulness showed indirect associations with dispositional flow through cognitive reappraisal, sport confidence, and the sequential pathway involving cognitive reappraisal and sport confidence.

### 5.2 Limitations and future directions

Although this study provides evidence for the association between sport-specific mindfulness and dispositional flow, as well as the independent and sequential indirect effects through cognitive reappraisal and sport confidence, several limitations should be noted.

(1) The cross-sectional questionnaire design limited our ability to draw conclusions about temporal ordering and causal direction. Although we conducted sensitivity analyses using alternative PROCESS Model 6 specifications, the comparable total indirect effects across forward and reverse models were consistent with the mathematical equivalence of cross-sectional mediation models and did not provide evidence that one temporal ordering was superior to another. We therefore interpreted the proposed model as a theory-guided representation of the observed associations. Future longitudinal or experimental studies are needed to further examine the temporal sequence among sport-specific mindfulness, cognitive reappraisal, sport confidence, and dispositional flow.

(2) Cognitive reappraisal was assessed using the ERQ cognitive reappraisal subscale, which primarily reflects a general tendency to use cognitive reappraisal and does not directly capture reappraisal during training or competition. As the other main constructs were assessed in sport-related contexts, this measurement-context difference may have attenuated the associations involving cognitive reappraisal and may have resulted in conservative estimates of the corresponding indirect effects. Future studies could extend the present findings by using or developing sport-specific measures of cognitive reappraisal.

(3) We recruited participants through convenience sampling from selected universities in Guangxi, Hebei, Liaoning, and Tianjin, which may limit the generalizability of the findings. We also did not collect detailed sport-specific characteristics, such as sport type, competitive level, training volume, or competition frequency. This limitation should be considered when interpreting the findings, as dispositional flow may differ across sport contexts, including individual versus team sports and closed-skill versus open-skill sports. Without information on sport-type distribution, we could not determine whether the observed associations reflected a general pattern among collegiate athletes or were partly influenced by particular sport categories. Unmeasured sport-type and training-related differences may also have confounded or moderated the observed mediation effects. Future studies should collect detailed sport-related characteristics and examine whether the proposed pathways differ across sport types, competitive levels, and training backgrounds.

(4) This study relied on self-report questionnaires and focused primarily on psychological variables. Although common method bias was assessed using both Harman’s single-factor test and the ULMC approach, potential self-report bias and common method bias cannot be completely ruled out. In addition, objective sport-performance or behavioral indicators were not included. Future research could combine self-report data with objective performance or behavioral measures and sport-context variables to construct a more comprehensive model of dispositional flow in collegiate athletes.

## Data Availability

The raw data supporting the conclusions of this article will be made available by the authors, without undue reservation.

## References

[B1] Amemiya R. Kim E. Inagaki K. Sakairi Y. (2019). Mindfulness and psychological competitive abilities promotes the mental health of athletes. *Japanese J. Sport Psychol.* 46, 67–80. 10.4146/jjspopsy.2019-1802

[B2] Arora A. (2025). Mindfulness and cognitive flexibility: A review of behavioral and neural evidence. *Int. J. Psychol. Stud.* 17:15. 10.5539/ijps.v17n3p15

[B3] Bandura A. (2001). Social cognitive theory: An agentic perspective. *Annu. Rev. Psychol.* 52, 1–26. 10.1146/annurev.psych.52.1.1 11148297

[B4] Baumgartner N. Wang C. H. Pritt T. Kao S. C. (2022). The acute effect of mindful high-intensity interval training on inhibitory control, attention, and cognitive flexibility. *Med. Sci. Sports Exerc.* 54, 59–60. 10.1249/01.mss.0000875772.48442.b4

[B5] Birrer D. Scalvedi B. Frings N. (2023). A bibliometric analysis of mindfulness and acceptance research in sports from 1969 to 2021. *Mindfulness* 14, 1038–1053. 10.1007/s12671-023-02124-5

[B6] Cepeda Salas V. M. Romero Carrasco A. E. (2014). El deportista y la atención plena / The athlete and mindfulness. *Revista Liminales. Escritos sobre Psicol. Soc.* 3, 173–187. 10.54255/lim.vol3.num05.247

[B7] Chang W. H. Chang J. H. Chen L. H. (2018). Mindfulness enhances change in athletes’ well-being: The mediating role of basic psychological needs fulfillment. *Mindfulness* 9, 815–823. 10.1007/s12671-017-0821-z

[B8] Contreras Pérez V. Crobu R. (2018). El mindfulness como intervención en psicología del deporte: Revisión sistemática. *Rev. Psicol. Aplicada Deporte Ejercicio Físico* 3, 1–15. 10.5093/rpadef2018a14

[B9] Cox A. E. Ullrich-French S. Austin B. (2020). Testing the role of state mindfulness in facilitating autonomous physical activity motivation. *Mindfulness* 11, 1018–1027. 10.1007/s12671-020-01311-y

[B10] Farb N. A. S. Anderson A. K. Segal Z. V. (2012). The mindful brain and emotion regulation in mood disorders. *Can. J. Psychiatry* 57, 70–77. 10.1177/070674371205700203 22340146 PMC3303604

[B11] Gardner F. L. Moore Z. E. (2012). Mindfulness and acceptance models in sport psychology: A decade of basic and applied scientific advancements. *Can. Psychol.* 53, 309–318. 10.1037/a0030220

[B12] Gilson T. A. (2010). Outcomes of confidence in sport training settings. *Strength Conditioning J.* 32, 91–96. 10.1519/ssc.0b013e3181dfcab7

[B13] Gross J. J. John O. P. (2003). Individual differences in two emotion regulation processes: Implications for affect, relationships, and well-being. *J. Pers. Soc. Psychol.* 85, 348–362. 10.1037/0022-3514.85.2.348 12916575

[B14] Guo J. Z. (2025). The relationship between mindfulness and flow state in athletes. *J. Educ. Human. Soc. Sci.* 53, 7–12. 10.54097/7f66m794

[B15] Hadash Y. Dar O. Amir I. Braver T. S. Bernstein A. (2026). The Mindfulness internal attention (MIA) framework: Uncovering the attentional mechanisms of mindfulness training. *Annu. Rev. Psychol.* 77, 255–283. 10.1146/annurev-psych-012925-030843 40758951

[B16] Jadhav R. (2025). The role of mindfulness in reducing anxiety and enhancing athletic performance. *Int. J. Multidisciplinary Res.* 7:54134. 10.36948/ijfmr.2025.v07i04.54134

[B17] Jekauc D. Fiedler J. Wunsch K. Mülberger L. Burkart D. Kilgus A. et al. (2025). The effect of self-confidence on performance in sports: A meta-analysis and narrative review. *Int. Rev. Sport Exerc. Psychol.* 18, 345–371. 10.1080/1750984x.2023.2222376

[B18] Kuloor H. (2024). “Self-confidence and sports performance,” in *Recent Research Advances in Arts and Social Studies*, Vol. 7, ed. R. C. Rus (Bhanjipur: BP International), 77–83. 10.9734/bpi/rraass/v7/7561b

[B19] Lindsay E. K. Creswell J. D. (2017). Mechanisms of mindfulness training: Monitor and acceptance theory (MAT). *Clin. Psychol. Rev.* 51, 48–59. 10.1016/j.cpr.2016.10.011 27835764 PMC5195874

[B20] Liu W. Liu X. Ji L. Watson J. C. Zhou C. Yao J. (2012). Chinese translation of the Flow state scale-2 and the dispositional flow scale-2: Examination of factorial validity and reliability. *Int. J. Sport Psychol.* 43, 153–175. 10.7352/ijsp.2012.43.153

[B21] Lu L. D. Heinze K. L. Soderstrom S. (2018). Playing multiple positions: Student-athlete identity salience and conflict. *J. Intercollegiate Sport* 11, 214–241. 10.1123/jis.2018-0034

[B22] Mahalingam R. (2022). “Mindfulness, competition, and sports psychology: A phenomenological perspective,” in *The Oxford Handbook of the Psychology of Competition*, eds Garcia S. M. Tor A. Elliot A. J. (Oxford: Oxford University Press), 598–610. 10.1093/oxfordhb/9780190060800.013.25

[B23] Noetel M. Ciarrochi J. Van Zanden B. Lonsdale C. (2017). Mindfulness and acceptance approaches to sporting performance enhancement: A systematic review. *Int. Rev. Sport Exerc. Psychol.* 12, 139–175. 10.1080/1750984x.2017.1387803

[B24] Pidgeon A. M. O’Brien B. Hanna A. Klaassen F. (2014). Cultivating a resilient response to stress through mindfulness and cognitive re-appraisal: A pilot randomised control trial. *GSTF J. Psychol.* 1, 8–13. 10.5176/2345-7872_1.2.13

[B25] Putri S. A. W. Handayani A. (2020). Hubungan antara kepercayaan diri dan regulasi emosi terhadap kecemasan menghadapi pertandingan pada atlet karate di Kota Demak. *Proyeksi* 15, 88–97. Indonesian. 10.30659/jp.15.1.88-97

[B26] Qin Y. Ren Z. Wu J. Liu B. Lin X. Yang Y. et al. (2022). The effects of mindfulness interventions on self-efficacy: A systematic review and meta-analysis. *Med. Sci. Sports Exerc.* 54, 466–467. 10.1249/01.mss.0000880916.02031.b5

[B27] Quaglia J. T. Zeidan F. Grossenbacher P. G. Freeman S. P. Braun S. E. Martelli A. et al. (2019). Brief mindfulness training enhances cognitive control in socioemotional contexts: Behavioral and neural evidence. *PLoS One* 14:e0219862. 10.1371/journal.pone.0219862 31323050 PMC6641506

[B28] Radford K. (2024). “Conservation of resources theory,” in *A Guide to Key Theories for Human Resource Management Research*, eds Hutchings K. Michailova S. Wilkinson A. (Cambridge, MA: Edward Elgar Publishing), 59–66. 10.4337/9781035308767.ch05

[B29] Regborn F. F. Holmström S. Svensson M. Sjögren M. (2025). Emotion regulation and mental health in young elite athletes. *Sports* 13:284. 10.3390/sports13090284 41003590 PMC12473469

[B30] Robazza C. Morano M. Bortoli L. Ruiz M. C. (2023). Athletes’ basic psychological needs and emotions: The role of cognitive reappraisal. *Front. Psychol.* 14:1205102. 10.3389/fpsyg.2023.1205102 37519370 PMC10374325

[B31] Rogowska A. M. Tataruch R. (2024). The relationship between mindfulness and athletes’ mental skills may be explained by emotion regulation and self-regulation. *BMC Sports Sci. Med. Rehabil.* 16:68. 10.1186/s13102-024-00863-z 38504372 PMC10949773

[B32] Scott-Hamilton J. Schutte N. S. Brown R. F. (2016). Effects of a mindfulness intervention on sports-anxiety, pessimism, and flow in competitive cyclists. *Appl. Psychol. Health Well-Being* 8, 85–103. 10.1111/aphw.12063 26970111

[B33] Simione L. Saldarini F. (2023). A critical review of the monitor and acceptance theory of mindfulness. *Mindfulness* 14, 1317–1328. 10.1007/s12671-023-02129-0

[B34] Stover A. D. Shulkin J. Lac A. Rapp T. (2024). A meta-analysis of cognitive reappraisal and personal resilience. *Clin. Psychol. Rev.* 110:102428. 10.1016/j.cpr.2024.102428 38657292

[B35] Sukmana E. D. Rianto A. Kauli I. Salmin S. Susilo R. K. D. Sutawi S. et al. (2025). The role of mindfulness in enhancing athletic performance: A critical review of empirical studies. *Retos* 69, 1295–1309. 10.47197/retos.v69.116706

[B36] Usher E. L. Morris D. B. (2023). “Self-efficacy,” in *Encyclopedia of Mental Health*, 3rd Edn, eds Friedman H. S. Markey C. H. (Amsterdam: Elsevier), 117–124. 10.1016/b978-0-323-91497-0.00085-0

[B37] Vealey R. S. Knight B. J. (2002). “Multidimensional sport-confidence: a conceptual and psychometric extension,” in *Paper presented at the Association for the Advancement of Applied Sport Psychology Conference*, (Tucson, AZ).

[B38] Wenzel M. Rowland Z. Kubiak T. (2021). Like clouds in a windy sky: Mindfulness training reduces negative affect reactivity in daily life in a randomized controlled trial. *Stress Health* 37, 232–242. 10.1002/smi.2989 32979027

[B39] Xu G. Yang X. Zhang Q. Li C. (2025). The impact of athlete burnout on academic burnout among college athletes: A multiple mediation model based on emotion regulation and sleep. *Front. Psychol.* 16:1669344. 10.3389/fpsyg.2025.1669344 41035448 PMC12479255

[B40] Young R. D. Neil E. R. Eberman L. E. Armstrong T. A. Winkelmann Z. K. (2023). Experiences of current National Collegiate Athletic Association Division I collegiate student-athletes with mental health resources. *J. Athletic Train.* 58, 704–714. 10.4085/1062-6050-0180.22 35788678 PMC11215735

[B41] Zhang C. Q. Chung P. K. Si G. (2017). Assessing acceptance in mindfulness with direct-worded items: The development and initial validation of the Athlete Mindfulness Questionnaire. *J. Sport Health Sci.* 6, 311–320. 10.1016/j.jshs.2015.09.010 30356584 PMC6189008

[B42] Zhou S. Wu Y. Xu X. (2023). Linking cognitive reappraisal and expressive suppression to mindfulness: A three-level meta-analysis. *Int. J. Environ. Res. Public Health* 20:1241. 10.3390/ijerph20021241 36673984 PMC9859341

