# Supplementary material for: The effect of sport-specific mindfulness on dispositional flow in collegiate athletes: the sequential mediating roles of cognitive reappraisal and sport confidence
Source: Front Psychol. 2026 Sep 24;17:1859598. doi: 10.3389/fpsyg.2026.1859598 (PMC13645538; doi:10.3389/fpsyg.2026.1859598)
Supplement: Supplementary file 1 [file Table_1.docx]

**Table S1. Sensitivity analyses of competing PROCESS Model 6 specifications**

| **Model** | **Path direction** | **Specific indirect effect 1** | **Specific indirect effect 2** | **Sequential indirect effect** | **Total indirect effect** |
| --- | --- | --- | --- | --- | --- |
| Proposed model | Sport-specific mindfulness → Cognitive reappraisal → Sport confidence → Dispositional flow | Sport-specific mindfulness → Cognitive reappraisal → Dispositional flow = 0.113 | Sport-specific mindfulness → Sport confidence → Dispositional flow = 0.019 | Sport-specific mindfulness → Cognitive reappraisal → Sport confidence → Dispositional flow = 0.012 | 0.144 |
| Reversed mediator-order model | Sport-specific mindfulness → Sport confidence → Cognitive reappraisal → Dispositional flow | Sport-specific mindfulness → Sport confidence → Dispositional flow = 0.031 | Sport-specific mindfulness → Cognitive reappraisal → Dispositional flow = 0.098 | Sport-specific mindfulness → Sport confidence → Cognitive reappraisal → Dispositional flow = 0.015 | 0.144 |
| Reverse X/Y model | Dispositional flow → Cognitive reappraisal → Sport confidence → Sport-specific mindfulness | Dispositional flow → Cognitive reappraisal → Sport-specific mindfulness = 0.101 | Dispositional flow → Sport confidence → Sport-specific mindfulness = 0.017 | Dispositional flow → Cognitive reappraisal → Sport confidence → Sport-specific mindfulness = 0.008 | 0.126 |
| Reverse X/Y + reversed mediators model | Dispositional flow → Sport confidence → Cognitive reappraisal → Sport-specific mindfulness | Dispositional flow → Sport confidence → Sport-specific mindfulness = 0.025 | Dispositional flow → Cognitive reappraisal → Sport-specific mindfulness = 0.089 | Dispositional flow → Sport confidence → Cognitive reappraisal → Sport-specific mindfulness = 0.012 | 0.126 |

Note. All variables were analyzed using standardized item-average scores. All models controlled for sex, age group, and years of sport participation. Values represent standardized indirect effects. The analyses were conducted to examine the robustness of the indirect-effect pattern across alternative mediator orders and predictor-outcome specifications.
